# MoS_2_-Cu/CuO@graphene Heterogeneous Photocatalysis for Enhanced Photocatalytic Degradation of MB from Water

**DOI:** 10.3390/polym14163259

**Published:** 2022-08-10

**Authors:** Asim Jilani, Ammar A. Melaibari

**Affiliations:** 1 Center of Nanotechnology, King Abdulaziz University, Jeddah 21589, Saudi Arabia; 2 Department of Mechanical Engineering, King Abdulaziz University, Jeddah 21589, Saudi Arabia

**Keywords:** photocatalysis, dye degradation, MoS_2_, graphene supported catalysts, water cleaning

## Abstract

The industrial revolution resulted in the contamination of natural water resources. Therefore, it is necessary to save and recover the natural water resources. In this regard, polymer-based composites have attracted the scientific community for their application in wastewater treatment. Herein, molybdenum disulfide composites with a mix phase of copper, copper oxide and graphene (MoS_2_-Cu/CuO@GN) were synthesized through the hydrothermal method. Methylene blue (MB) was degraded by around 93.8% within the 30 min in the presence of MoS_2_-Cu/CuO@GN under visible light. The degradation efficiency was further enhanced to 98.5% with the addition of H_2_O_2_ as a catalyst. The photocatalytic degradation efficiency of pure MoS_2_, MoS_2_-Cu/CuO and MoS_2_-Cu/CuO@GN were also investigated under the same experimental conditions. The structural analysis endorses the presence of the Cu/CuO dual phase in MoS_2_. The charge recombination ratio and band gap of MoS_2_-Cu/CuO@GN were also investigated in comparison to pure MoS_2_ and MoS_2_-Cu/CuO. The chemical states, the analysis of C1s, O1s, Mo3d and Cu2p3, were also analyzed to explore the possible interaction among the present elements. The surface morphology confirms the existence of Cu/CuO and GN to MoS_2_.

## 1. Introduction

Environmental and energy remediation are two major issues for human beings due to rapid industrialization [1]. The rapid increase in industrialization activities resulted in the contamination of natural water resources. The organic dyes and various kinds of heavy metals are being discharged into water reservoirs on a daily basis from different industrial activities [2]. These contaminations in water are a severe threat for human health and the ecosystem. In this regard, various materials and methods have been deployed to remove the contamination from the water, such as absorption, membrane filtration and photocatalytics [3]. However, the photocatalytic process is being considered an efficient and cost-effective way to remove or neutralize hazardous material from water [4]. Therefore, various materials such as carbon-based metals and metals oxides and, more importantly, polymers are attractive candidates to remove the pollutants through the photocatalytic process [5].

Polymers are among the most versatile materials for wastewater treatment through photocatalysts, membranes and the absorption process owing to chemical stability/versatility, ease of functionalization, high specific surface, etc., [6,7]. Therefore, a two-dimensional-layered structure of molybdenum disulfide (MoS_2_) has attracted the scientific community because of its extraordinary structure and properties such as strong oxidizing activity, non-toxicity in nature and a low band gap (1.8 eV) that can be further tuned with quantum confinement effects [8]. This low band gap of the MoS_2_ is beneficial to absorb the light photons in the visible region which is highly desirable to enhance the photocatalytic process [9,10]. However, the interaction of Mo–S can engender the unsaturated atoms at the crystal edge which may render the photocatalytic activity of MoS_2_ [11]. Therefore, the doping of some metals or metal oxides can enhance the photocatalytic activity of MoS_2_.

In this context, p-type semiconductor materials such as copper oxide (CuO) have gained attention due to their photo-conductivity nature, which supports enhancing the photocatalytic activity of n-type MoS_2_ by reducing the charge recombination ratio [12]. Moreover, CuO doping can enhance the absorption of light photons which results in the augmentation of catalytic activity [13]. Further the mix phase of Cu/CuO supports the formation of heterojunction which reduces the recombination of charge carriers that can enhance the photocatalytic activity [14]. However, the access amount of CuO in composites can provide the recombination center for charge carriers which will reduce the photocatalytic activity [15]. Therefore, the optimal amount of CuO is also important to enhance photocatalytic activity.

The charge carrier separation can be enhanced by the addition of graphene (GN) which amended the charge separation and absorbed the more visible light which consequently enhanced the photocatalytic activity [16]. Moreover, GN can also create some defects while making the composites with metals and metal oxides. These defects can capture the pollutant during the photocatalytic process [17] which makes it attractive for wastewater treatment. Further, the high specific surface area (2650 m^2^/g), π orbital, π–π interaction, functional groups makes it an attractive dopant with metal, metal oxides and polymers to synthesiss the photocatalysts [18].

Previous studies show that there are various reports on MoS_2_ with GN and CuO and other metal oxides as a catalyst [12,19,20]. However, the low catalytic performance is still a major issue for these binary composites. Further, there are few reports on the role of metals and metal oxides in the photocatalytic activity of MoS_2_. Therefore, in this study, the combination of MoS_2_ with GN and Cu/CuO mix phases were synthesized through an in-suit hydrothermal process. The role of Cu/CuO and GN to enhance the photocatalytic activity of MoS_2_ was explored. Moreover, the change in the functional groups through XPS and the change in optical and structural properties were also studied.

## 2. Experimental Section

### 2.1. Materials

Molybdenum (VI) oxide (99.97%), thiourea (≥99.0%), copper (II) sulfate (≥99.99%), CTAB (≥99.0%), ascorbic acid (≥99.0%), sodium hydroxide (≥98.0%), graphite powder (≥99.99%), sulfuric acid (95 to 98%) and methylene blue (80%) were purchased from Sigma Aldrich Burlington USA and used for the synthesis of MoS_2_-Cu/CuO@GN without any further treatment.

### 2.2. Synthesis of MoS_2_-Cu/CuO@GN

MoS_2_ was synthesized by hydrothermal methodology using MoO_3_ and thiourea as precursors. In a typical process, 0.1150 g of MoO_3_ and 0.2664 g of thiourea was taken in 80 mL of water and the system was put under stirring conditions for 30 min. Thereafter, the whole reaction mixture was transferred to 100 mL of Teflon-lined hydrothermal reactor and subsequently heated at 200 °C for 24 hrs. Thus, obtained the black precipitate of MoS_2_ was separated by centrifugation, washed with excess of water and ethanol, dried at 80 °C for 12 h and subsequently stored in desiccator for further experiments.

For the MoS_2_-Cu/CuO@GN, first, binary composite of MoS_2_-Cu/CuO was prepared and further coating of GN over it resulted in MoS_2_-Cu/CuO@GN. The GO (stock solution of 10 mg/mL) and Cu/CuO nanoparticles were prepared separately. The synthesis of GO can be seen elsewhere [21]. The Cu/CuO nanoparticles were synthesized by the reduction of copper (II) sulfate in the presence of CTAB surfactant. In a typical process, 0.1 M copper (II) sulfate solution was dissolved in 100 mL of water and to it, 0.25 g of CTAB was added and the whole system was put under stirring conditions. In another beaker, 50 mL of 0.2 M ascorbic acid solution was prepared. In the second step, the solution of ascorbic acid was slowly added to the copper (II) sulfate solution and, subsequently, 30 mL of 1 M sodium hydroxide solution was also added. The whole system was heated to 80 °C for 2 h and a dark reddish–brown color confirmed the formation of Cu/CuO. Thus, prepared Cu/CuO was separated by centrifugation, washed with excess of water and ethanol and subsequently dried at room temperature [22]. The MoS_2_-Cu/CuO was prepared by mixing 1 g of MoS_2_ and 0.1 g of Cu/CuO in 50 mL ethanol and the mixture was put in ultrasonic bath for 1 h, followed by stirring on hot plate until the complete evaporation of ethanol. Further, the fabrication of ternary MoS_2_-Cu/CuO@GN was done by mixing 10 mL of GO with 1 g of MoS_2_-Cu/CuO and the whole mixture was heated at 400 °C for 3 h for the complete reduction of GO into GN and to, subsequently, give MoS_2_-Cu/CuO@GN. The ratios of MoS_2_, Cu/CuO and GN were, respectively, 87.1%, 8.71 and 4.19%.

### 2.3. Photodegradation Measurement

MB was selected as model pollutant to assess the catalytic performance of MoS_2_, MoS_2_-Cu/CuO and MoS_2_-Cu/CuO@GN. In this regard, 25 mg of catalyst (optimized against initial concentration of MoS_2_ and pH Figure S1) was added to the 20 ppm aqueous solution of MB. However, the MB solution containing the catalyst was put in the dark under vigorous stirring for 30 min to achieve the adsorption desorption equilibrium. Afterwards, the solution was irradiated with visible light of 2 watt, having a distance of 12 cm from MB solution for 30 min. The intensity was approximately 11.06 watt/meter. During this irradiation, a certain amount (5 mL) of solution was taken to estimate the degradation of MB by measuring the spectrum through UV-Visible spectrometer.

The MB degradation ability of prepared photocatalysts was calculated by applying the following relation [23]:

(1) $$Degradation\;\left(\%\right)=\left(\frac{C_{o}-C_{t}}{C_{o}}\right)100$$

where *C_o_* represent the initial taken concentration of MB while *C_t_* symbolizes the remaining MB concentration after interval of 10 min. Once the photocatalytic efficiency was calculated, then following giving relation was used to calculate the reaction rate constant during degradation process [24].

(2) $$\ln(\frac{C}{C_{o}})=\;-kt$$

### 2.4. Characterizations

The structural and surface compositional analysis of MoS_2_, MoS_2_-Cu/CuO and MoS_2_-Cu/CuO@GN were performed, respectively, with X-ray diffraction (Ultima IV-Rigaku Tokyo Japan) and X-ray photoelectron spectroscopy (PHI-Versa ProbeII Chanhassen USA). The pass energies of 187.85 eV and 47.46.95 eV were used, respectively, to acquire the survey and narrow scan mode. Surface morphology was investigated by field emission scanning electron microscopy (JSM7600-F-Jeol Tokyo Japan). The spectrometer (DR 6000 Hach Loveland USA) was used to calculate the absorption of MB, while charge recombination ratio was investigated through photoluminescence spectrometer (Shimadzu RF 5301PC Kyoto Japan).

## 3. Results and Discussion

### 3.1. Structural Analysis

**Figure 1:**
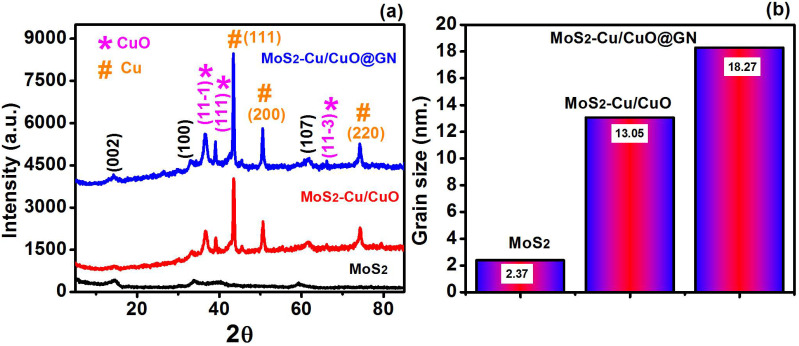
Diffraction analysis (**a**) and grain size calculation (**b**) for MoS_2_, MoS_2_-Cu/CuO and MoS_2_-Cu/CuO@GN.

The diffraction analysis of MoS_2_, MoS_2_-Cu/CuO and MoS_2_-Cu/CuO@GN (Figure 1) reveals the clear diffraction peaks which are attributed to the crystalline nature of synthesized photocatalysts. The diffraction pattern of pure MoS_2_ revealed the diffraction peak around 2θ = 14.27, 33.04 and 61.65 which, respectively, attributed to the diffraction planes, (002), (100) and (1007), of the molybdenite-2H phase (JCPD #00-037-1492). The addition of Cu/CuO to MoS_2_ (MoS_2_-Cu/CuO) resulted in enhancing the sharp diffraction pattern with some additional new diffraction peaks in comparison to the pure MoS_2_ diffraction pattern. The additional diffraction appeared around 2θ = 36.59, 38.98, 43.43, 50.56, 61.65 and 74.20. The diffraction peaks at 2θ = 36.59, 38.98 and 61.65 are attributed to the tenorite phase of CuO (JCPD # 00-001-1117), while the diffraction peaks at 2θ = 43.43, 50.56 and 74.20 are the representation of copper (Cu), as revealed in JCPD # 01-085-1326. Therefore, XRD analysis confirms the presence of Cu/CuO with MoS_2_. Moreover, after the addition of GN (MoS_2_-Cu/CuO@GN), no additional peak was observed. The non-observable diffraction peak of GN is attributed due to the exfoliation nature [25]. Further, the functional groups of GN can interact with MoS_2_ and Cu/CuO may lead to variations in the structural properties without changing the preferred orientation of the diffraction planes [26]. This could be noticed in our diffraction analysis (Table 1) that shows the change in the crystal grain size, which also revealed the successful interaction of GN functional groups with MoS_2_ and Cu/CuO. The Scherrer relation was used to estimate the crystal size of MoS_2_, MoS_2_-Cu/CuO and MoS_2_-Cu/CuO@GN [27].

(3) $$D=\frac{K\lambda }{\beta cos\theta }$$

The crystal grain size of MoS_2_ was around 2.37 nm, while it was increased to 13.05 and 18.27, respectively, for MoS_2_-Cu/CuO and MoS_2_-Cu/CuO@GN. This increment in the crystal grain size has a direct relation to the enhancement of the photocatalytic activity of the material, as reported previously [28]. It could also be noticed that (Section 3.5) MoS_2_-Cu/CuO@GN enhanced the photocatalytic activity in comparison to MoS_2_ and MoS_2_-Cu/CuO. The interaction of Cu/CuO and GN can further lead to a change in the dislocation density of MoS_2_ and be calculated by the following equation [29].

(4) $$\delta =\frac{1}{D^{2}}$$

The dislocation density was calculated around 4.43 × 10^−1^, 1.81 × 10^−2^ and 1.96 × 10^−2^, respectively, for MoS_2_, MoS_2_-Cu/CuO and MoS_2_-Cu/CuO@GN. This variation in the location density further leads to a change in the lattice strain of MoS_2_ after the addition of Cu/CuO and GN. This change was calculated by the following relation [30].

(5) $$\varepsilon =\frac{\beta cos\theta }{4}$$

The lattice strain was around 2.01 × 10^−2^, 4.00 × 10^−3^ and 3.79 × 10^−3^, respectively, for MoS_2_, MoS_2_-Cu/CuO and MoS_2_-Cu/CuO@GN. In summary, the structural analysis (Table 1) showed the change in the lattice parameter of MoS_2_ after the addition of Cu/CuO and GN. However, this addition does not lead to a change in the diffraction orientation or phase of MoS_2_.

### 3.2. Optical Properties

**Figure 2:**
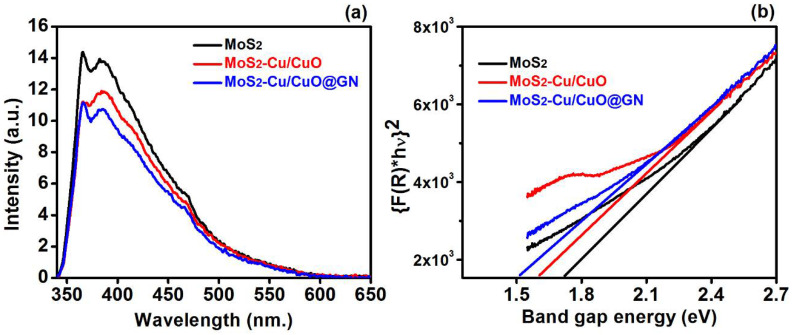
PL analysis (**a**) and band gap calculation (**b**) for MoS_2_, MoS_2_-Cu/CuO and MoS_2_-Cu/CuO@GN.

The surface oxygen defects and charge recombination in the photocatalytic material can affect the catalytic efficiency which can be estimated by the PL spectra. Moreover, the peak intensity of PL spectra is attributed to the charge recombination during charge propagation from the valence to conduction band. The PL spectra (Figure 2a) of MoS_2_, MoS_2_-Cu/CuO and MoS_2_-Cu/CuO@GN was recorded from 350 to 650 nm, having the 320 nm excitation wavelength. The PL intensity of the MoS_2_ is higher in comparison to MoS_2_-Cu/CuO and MoS_2_-Cu/CuO@GN, which revealed the higher recombination rate of the charged carrier in MoS_2_. However, the intensity of MoS_2_ reduced after the addition of Cu/CuO (i.e., MoS_2_-Cu/CuO), which indicated the role of Cu/CuO for charge separation and transfer at the heterojunction of MoS_2_-Cu/CuO [31]. Moreover, the decrease in the PL intensity of MoS_2_-Cu/CuO was also associated to the chemisorption absorption of the oxygen over the surface of the catalyst, which resulted in the enhanced charge separation [32]. PL intensity was further reduced for MoS_2_-Cu/CuO@GN and this lessening is attributed to the interface between GN and MoS_2_-Cu/CuO. Moreover, functional groups attached to the basal planes of GN provided the attractive site to enhance the charge carrier movement by reducing the charge recombination, which ultimately enhanced the catalytic activity of the MoS_2_-Cu/CuO@GN [33].

The change in the recombination of the charge carrier can change the band gap of the MoS_2_, which ultimately affects the photocatalytic activity. Therefore, the band gap of MoS_2_, MoS_2_-Cu/CuO and MoS_2_-Cu/CuO@GN was estimated by applying the Kubelka–Munk relation [34]. The band gap of MoS_2_ (Figure 2b) was approximately 1.7 eV which is consistent with the previous literature [35]. The band gap of MoS_2_ reduced to 1.60 eV after the addition of Cu/CuO (MoS_2_-Cu/CuO). This reduction in the band gap is attributed to the absorption ability of Cu/CuO in the visible region, which results in the reduction of the MoS_2_-Cu/CuO band gap [36]. The band gap of MoS_2_-Cu/CuO@GN was approximately 1.50 eV, which enlightens the role of GN to reduce the band gap. Generally, the GN has an sp2 band and other oxygen functional groups in the form of epoxy (C-O-C) and hydroxyl (OH) which enhances the charge carrier mobility with the absorption of light in the GN-based composites [37].

### 3.3. Surface Compositional Analysis

**Figure 3:**
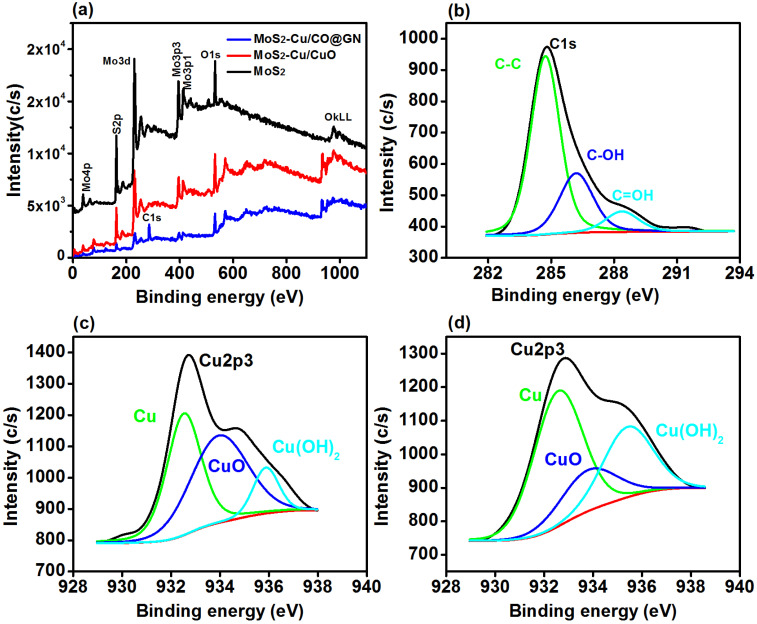
(**a**) Survey scan for MoS_2_, MoS_2_-Cu/CuO and MoS_2_-Cu/CuO@GN, (**b**) high resolution C1s spectra of MoS_2_-Cu/CuO@GN and (**c**,**d**) high resolution Cup2 analysis for MoS_2_-Cu/CuO and MoS_2_-Cu/CuO@GN.

The compositional analysis of MoS_2_, MoS_2_-Cu/CuO and MoS_2_-Cu/CuO@GN was acquired by the XPS survey scan. The surface composition (Figure 3a) of MoS_2_ revealed the presence of Mo3d, O1s and S2p with the atomic percentage of approximately 25.5%, 30.6 and 43.9%, respectively. There was no other element observed which shows the accuracy of the synthesis approach and is also consistent with our XRD diffraction pattern. The survey spectra of MoS_2_-Cu/CuO revealed the appearance of Cu2p3 in addition to Mo3d, O1s and S2p. Moreover, the incorporation of GN was confirmed by the presence of the C1s peak in MoS_2_-Cu/CuO@GN and was approximately 46.7%. Table 2 shows the detected atomic percentage of each detected element in the prepared catalyst.

The C1s spectra (Figure 3b) of MoS_2_-Cu/CuO@GN revealed the presence of three peaks approximately at 284.73 eV, 286.06 and 288.34 eV and attributed, respectively, to the C-C (65.82%), C-OH (25.44%) and C=O (8.74%) functional groups [38,39]. These functional groups of GN are important to facilitate/capture the dye molecules which, as a result, enhanced the catalytic activity of MoS_2_-Cu/CuO@GN [16].

Cu2p3 of MoS_2_-Cu/CuO (Figure 3c) revealed three peaks at 532.7 eV, 533.5 and 935.4 eV which are attributed to Cu, CuO and Cu (OH)_2_ [40,41]. Cu was approximately 53.13%, while CuO and Cu (OH)_2_ were, respectively, 18.70 and 28.17%. However, after the induction of GN (Figure 3d), the contribution of Cu changed from 53.13 to 42.41%, while CuO increased from 18.76 to 46.22%. In conclusion, the Cu2p3 analysis of MoS_2_-Cu/CuO and MoS_2_-Cu/CuO@GN confirms the presence of a mixed phase of Cu/CuO and is consistent with our XRD findings.

**Figure 4:**
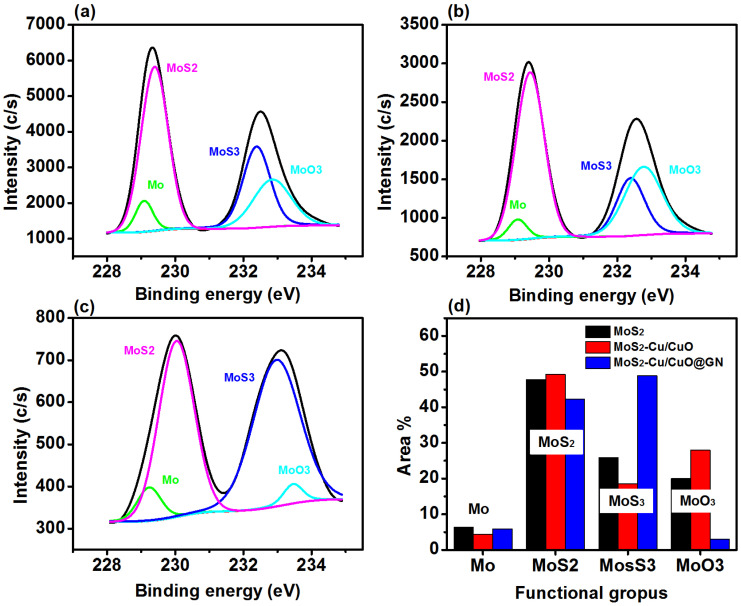
Mo3d spectra (**a**) MoS2, (**b**) MoS_2_-Cu/CuO, (**c**) MoS_2_-Cu/CuO@GN and (**d**) functional groups for MoS_2_, MoS_2_-Cu/CuO and MoS_2_-Cu/CuO@GN.

High-resolution spectra of Mo3d (Figure 4) show the four peaks which confirm the interaction of molybdenum with oxygen and sulfur. The MoS_2_ (Figure 4a) have peaks around 229.0 eV, 229.3, 232.5 and 232.8 eV which are, respectively, attributed to Mo, MoS_2_, MoS_3_ and MoO_3_ [41,42]. However, the Mo, MoS_2_, MoS_3_ and MoO_3_ contribution was changed with the addition of Cu/CuO and GN, as revealed in (Figure 4b–d). This change in the metal and oxide interaction can lead to changes in the photocatalytic activity of the catalyst [43].

**Figure 5:**
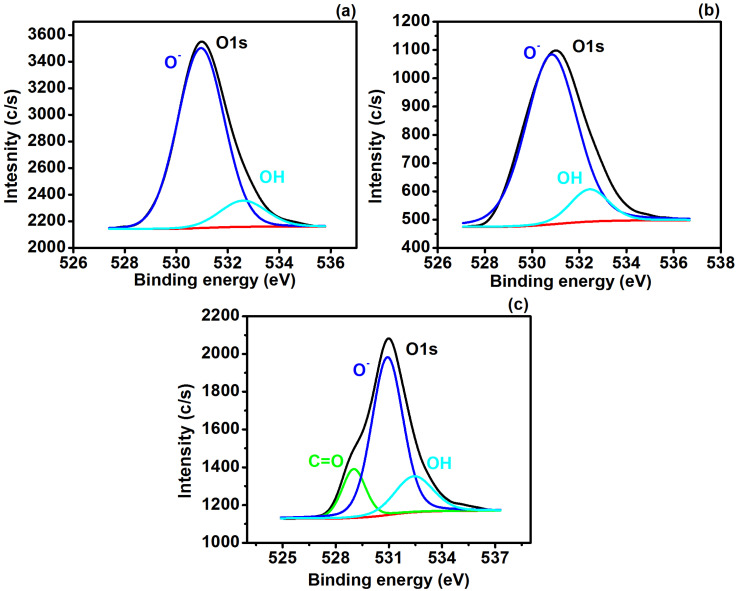
High-resolution O1s analysis for (**a**) MoS_2_, (**b**) MoS_2_-Cu/CuO and (**c**) MoS_2_-Cu/CuO@GN.

O1s spectra of MoS_2_ (Figure 5a) revealed the appearance of two peaks at approximately 530.9 and 532.5 eV which are attributed to the oxidation of O1s (O^−^) and OH [41,44]. However, the small shift in the contribution of O^−^ (87.08 to 87.55%) and OH (12.92 to 12.45%) were seen in the Cu/CuO counterpart (Figure 5b), which could be due to possible interactions among the constituent elements. The addition of GN to MoS_2_-Cu/CuO resulted in an additional peak (Figure 5c) at approximately 529.02 eV which presents the C–O bonding with the contribution of 15.41% [41].

### 3.4. Surface Morphology

**Figure 6:**
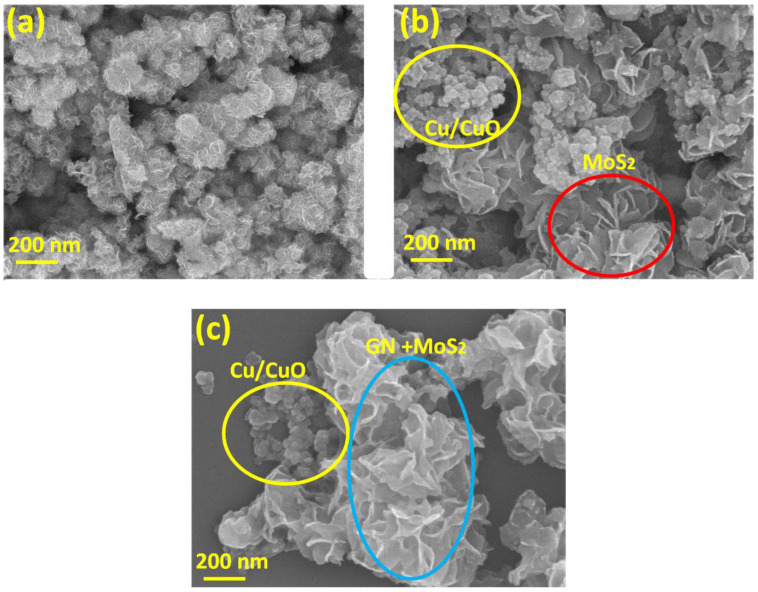
Surface morphology of (**a**) MoS_2_, (**b**) MoS_2_-Cu/CuO and (**c**) MoS_2_-Cu/CuO@GN.

FESEM images of MoS_2_ (Figure 6a) shows the stacked petal-like structure of MoS_2_ which is also consistent with previous reports [45]. The surface morphology of MoS_2_-Cu/CuO (Figure 6b) shows the appearance of some clusters of nanoparticles in addition to the stacked petal-like structure of MoS_2_ which are the Cu/CuO nanoparticles. Further, with the addition of GN (Figure 6c), flakes were observed with clustered MoS_2_ which is due to the possible interactions of GN with MoS_2_ and Cu/CuO [46].

### 3.5. Photocatalytic Activity

**Figure 7:**
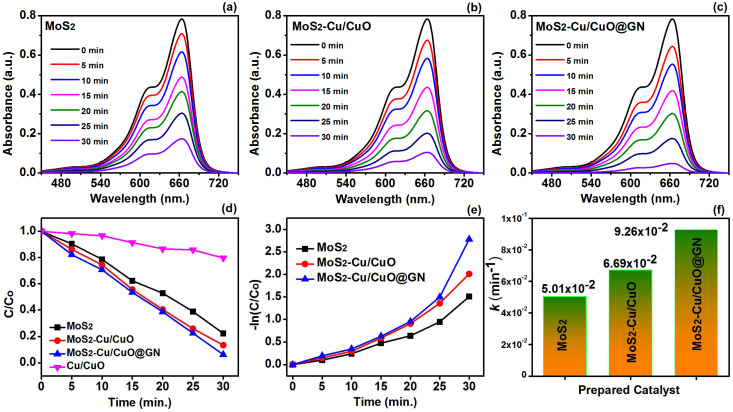
(**a**–**c**) Time-dependent dilapidation of the MB characteristic peak in the presence of Cu/CuO, MoS_2_, MoS_2_-Cu/CuO and MoS_2_-Cu/CuO@GN, (**d**,**e**) degradation percentage rate of MB within 30 min and (**f**) reaction rate constant of MoS_2_, MoS_2_-Cu/CuO and MoS_2_-Cu/CuO@GN.

The photocatalytic activity of the prepared MoS_2_, MoS_2_-Cu/CuO and MoS_2_-Cu/CuO@GN was tested against the degradation of MB. The UV abortion peak of MB appeared at 665 nm and was monitored for the purpose of degradation. Figure 7a–c show the time-dependent dilapidation of the MB characteristic peak in the presence of MoS_2_, MoS_2_-Cu/CuO and MoS_2_-Cu/CuO@GN, respectively. The intensity in Figure 7c was found lower in comparison to Figure 7a,b under the same experimental conditions, having the same concentration of MB. This lower intensity revealed a faster degradation rate. Figure 7d,e show the degradation percentage rate of MB within the 30 min for MoS_2_, MoS_2_-Cu/CuO and MoS_2_-Cu/CuO@GN. MoS_2_ and Cu/CuO degraded the MB around 77.8% and 20.4%, respectively, within 30 min under the visible light irradiation. However, the degradation ability of MoS_2_ accelerated after the addition of Cu/CuO and GN. Therefore, the degradation of MoS_2_-Cu/CuO and MoS_2_-Cu/CuO@GN was found to be 86.6 and 93.8%, respectively. The enhanced degradation ability of MoS_2_-Cu/CuO in comparison to MoS_2_ revealed the role of Cu/CuO to enhance the photocatalytic activity. This enhanced photocatalytic performance of MoS_2_-Cu/CuO attributed to the formation of a heterojunction, which ultimately enhanced the charge separation and slowed down the charge recombination ratio [47]. The photocatalytic activity of MoS_2_-Cu/CuO enhanced after the addition of GN. Therefore, MoS_2_-Cu/CuO@GN has a 93.8% efficiency for the degradation of MB within 30 min. This enhanced photocatalytic activity is also attributed to the surface area of the catalyst. The surface area of the MoS_2_ was found to be around 280.45 m^2^/g, while the surface area of MoS_2_-Cu/CuO and MoS_2_-Cu/CuO@GN was, respectively, 285.20 and 297.50 m^2^/g (Figure S2). This enhanced surface area also provides an ample active site to capture the dye molecules. Moreover, GN provided the support in electron transport and lessened the recombination of the charge carried, which resulted in the enhancement of the catalytic activity of MoS_2_-Cu/CuO@GN [48]. This change in the movement of the charge particle may affect the reaction rate constant (*k*) which is shown in Figure 7f. The order of the reaction rate constant was MoS_2_-Cu/CuO@GN > MoS_2_-Cu/CuO > MoS_2_ with values, respectively, of 5.01 × 10^−2^, 6.69 × 10^−2^ and 9.26 × 10^−2^.

#### 3.5.1. Effect of Reaction Parameter

##### Effect of MoS_2_-Cu/CuO@GN Dosage

**Figure 8:**
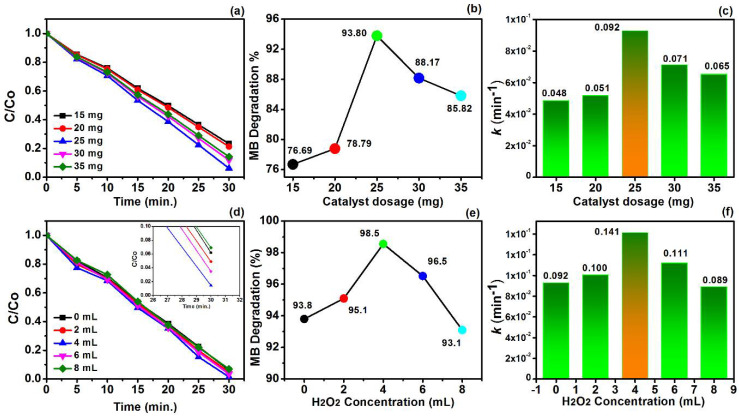
(**a**–**c**) Change in the reaction kinetics at different dosages of MoS_2_-Cu/CuO@GN, (**d**–**f**) change in the reaction kinetics at different dosages of hydrogen peroxide.

The degradation of MB can further be associated with the dosage concentration of MoS_2_-Cu/CuO@GN. The concentration of the catalyst played a vital role in producing the active site which assisted the degradation of the pollutant. However, an excess of active sites can render the catalytic performance. In this regard, the concentration of MoS_2_-Cu/CuO@GN was chosen from 15 to 35 mg, having an interval of 5 mg. The degradation efficiency was 76.69% at 15 mg/200 mL which further increased to 78.79 and 93.80, respectively, for 20 and 25 mg/200 mL, as shown in Figure 8a–c. This increasing trend in the degradation efficiency of MB with the increase in the MoS_2_-Cu/CuO@GN dosage revealed the ample active site on the surface of the prepared catalysts to capture and degrade the dye molecules. However, the catalytic efficiency was reduced to 88.17 (30 mg/200 mL) and 85.82% (35 mg/200 mL) with a further increase to the MoS_2_-Cu/CuO@GN dosage. This indicates that the blocking of the active sight and scattering of light radiation also affects the reaction rate constant (*k*) during the photocatalytic process.

##### Effect of Hydrogen Peroxide

The catalytic properties of the material can be tuned by adjusting the reaction parameters, such as by introducing the scavenging of free radicals. In this regard, H_2_O_2_ is commonly used for the production of reactive oxygen species such as hydroxyl radicals and superoxide during the photocatalytic reaction. These generated radicals can react with MB to enhance the photocatalytic efficiency. Therefore, we decided to investigate the photocatalytic activity of MoS_2_-Cu/CuO@GN at different concentrations of H_2_O_2_, i.e., 0, 2, 4, 6 and 8 mL. Figure 8d–f show the change in the reaction kinetics of MoS_2_-Cu/CuO@GN with the addition of H_2_O_2_. The degradation efficiency of MoS_2_-Cu/CuO@GN was approximately 93.8% in the absence of H_2_O_2_, having a rate constant of 0.092.min^−1^. However, the efficiency increased to 98.5% at 4% of H_2_O_2_, with the highest rate constant of 0.141 min^−1^. This revealed the generation of maximum free radicals that interacts with MB during the degradation process. However, the catalytic efficiency of MoS_2_-Cu/CuO@GN was reduced to 96.5% and 93.1 %, respectively, for 6 and 8mL of H_2_O_2_. This showed that free radicals react with H_2_O_2_ rather than MB which ultimately reduced the photocatalytic efficiency [49].

##### Reusability of MoS_2_-Cu/CuO@GN

**Figure 9:**
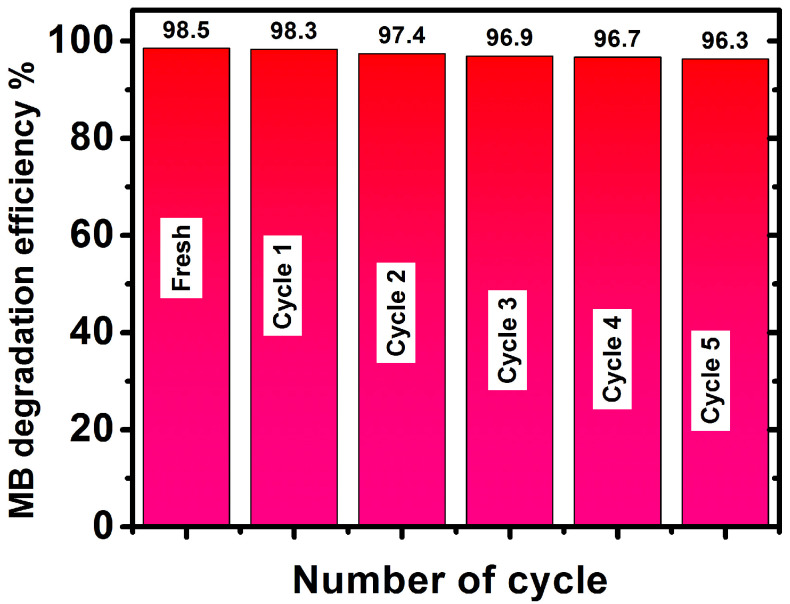
Reusability of MoS_2_-Cu/CuO@GN photocatalysts under same experimental conditions.

The reusability of the photocatalysts matters for their potential application. Therefore, the cyclic reusability of MoS_2_-Cu/CuO@GN was tested. The five consecutive cyclic photocatalytic experiments were performed. A total of 25 mg of MoS_2_-Cu/CuO@GN was added to 20 ppm of MB solution having 4% of H_2_O_2_. The sample was separated through a centrifuge (3000 rpm/min) after completing each cycle. The cyclic results (Figure 9) revealed that the photocatalytic efficiency remained at 96.3% after five consecutive cycles under the same experimental conditions.

## 4. Conclusions

In conclusion, the addition of Cu/CuO and GN accelerated the photocatalytic activity of MoS_2_ under certain optimized experimental conditions. This enhanced the photocatalytic activity of MoS_2_-Cu/CuO@GN, attributed to the change in the charge carrier movement, the alteration in the recombination ratio, the band gap, the interaction of the present element and the structural properties. The band gap of MoS_2_ reduced to 1.5 eV from 1.7 eV. Moreover, Cu/CuO and GN supports the chemisorption absorption of the oxygen over the surface of the catalyst by providing the more active sites, which resulted in the enhanced photocatalytic activity. The structural analysis revealed the increase in the grain size of MoS_2_ (2.37 nm) with the addition of Cu/CuO (13.05 nm) and GN (18.27 nm) without changing the preferred crystal orientation. The XPS analysis confirms the variation in the C-C, C-OH, C=O and OH functional groups of MoS_2_ with the addition of Cu/CuO and GN, which is attributed to the enhanced photocatalytic activity. In short, this study revealed the potential use of polymer-based nanocomposites with metal, metal oxides and graphene for wastewater treatment through a facile photocatalytic process.

## Figures and Tables

**Table 1:** Calculated structural parameters for MoS_2_, MoS_2_-Cu/CuO and MoS_2_-Cu/CuO@GN.

Material Details	Grain Size (nm)	Dislocation	Lattice Strain	Cell Volume Only for MoS_2_
MoS_2_	2.37	4.46 × 10^−1^	2.01 × 10^−2^	10.84
MoS_2_-Cu/CuO	13.05	1.81 × 10^−2^	4.00 × 10^−3^	10.96
MoS_2_-Cu/CuO@GN	18.27	1.96 × 10^−2^	3.79 × 10^−3^	10.47

**Table 2:** XPS Elemental composition of MoS_2_, MoS_2_-Cu/CuO and MoS_2_-Cu/CuO@GN.

Sample Detail	Detected Element (%)				
	S2p	O1s	Mo3d	Cu2P3	C1s
MoS_2_	43.9	30.6	25.5	---	----
MoS_2_ Cu/CuO	30.5	42.1	18.4	8.9	----
MoS_2_ Cu/CuO@GN	10.6	28.9	6.0	7.8	46.7

## Data Availability

Data will be available on request.

## Supplementary Materials

The following supporting information can be downloaded at: https://www.mdpi.com/article/10.3390/polym14163259/s1, Figure S1. (a) Degradation of MB under the different concentration MoS_2_ (b) Degradation of optimized 25 mg of MoS_2_ at various pH within the 30 minutes. Figure S2. BET specific surface area of MoS_2_, MoS_2_-Cu/CuO MoS_2_-Cu/CuO @GN.
