# Supplementary material for: MoS2-Cu/CuO@graphene Heterogeneous Photocatalysis for Enhanced Photocatalytic Degradation of MB from Water
Source: Polymers (Basel). 2022 Aug 10;14(16):3259. doi: 10.3390/polym14163259 (PMC9414561; doi:10.3390/polym14163259)
Supplement: Supplementary file 1 [file polymers-14-03259-s001.zip › polymers-1800215-supplementary.pdf]

MoS_2-Cu/CuO@graphene heterogeneous photocatalysis for enhanced photocatalytic degradation of MB from water

Asim Jilani [1]* Ammar A Melaibari [1, 2]

[1] Center of Nanotechnology, King Abdulaziz University, 21589, Jeddah, Saudi Arabia
[2] Department of Mechanical Engineering, King Abdulaziz University, Jeddah, Saudi Arabia
* Correspondence: asim.jilane@gmail.com, ajilani@kau.edu.sa

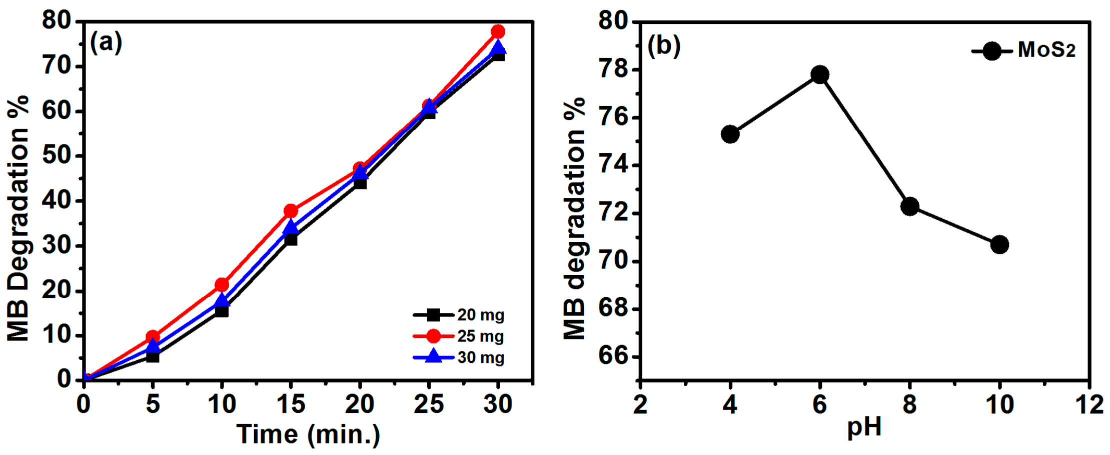

Figure S1. (a) Degradation of MB under the different concentration MoS_2 (b) Degradation of optimized 25 mg of MoS_2 at various pH within the 30 minutes

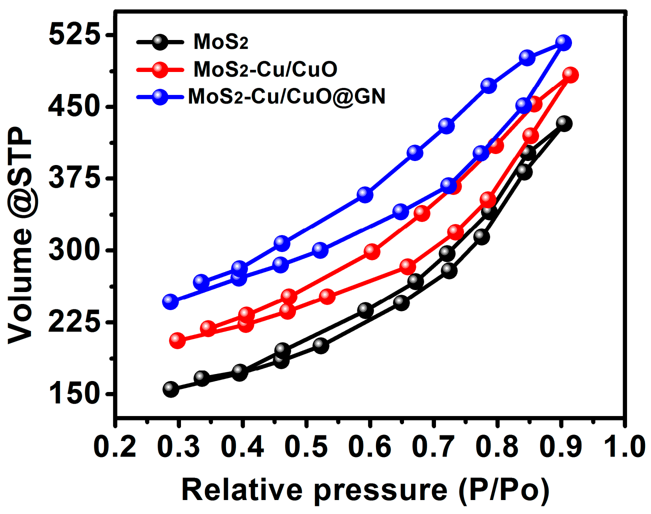

Figure S2. BET specific surface area of MoS_2 , MoS_2-Cu/CuO MoS_2-Cu/CuO @GN
